# ORGANIZING PRIMARY CARE FOR RESIDENTS IN LONG-TERM CARE FACILITIES: A MULTI-METHOD APPROACH

**DOI:** 10.1093/geroni/igz038.905

**Published:** 2019-11-08

**Authors:** Barbara Hanratty, Katie Brittain, Rachel Stocker, Karen Spilsbury

**Affiliations:** 1 Newcastle University, Newcastle upon Tyne, United Kingdom; 2 Northumbria university, Newcastle upon Tyne, England, United Kingdom; 3 University of Newcastle, newcastle upon Tyne, England, United Kingdom; 4 University of Leeds, Leeds, England, United Kingdom

## Abstract

This multi-method research explores the challenges family practitioners and long-term care facilities face when they work together. It seeks to understand how different responses to these challenges may influence the delivery of care. Whilst different services have their own values, aims, structures and processes, all are contending with constrained resources and frequent organisational change. Our findings from a large qualitative study and analysis of routine health data are organised around the micro (individual), meso (organisational) and macro (system) factors that influence the organisation and delivery of resident care. In this presentation, we draw out the interplay between these levels, and how each shapes and is shaped by the changing demands and nature of care. This presentation will bring new insights into primary care for long-term care facilities, through the perspectives of those who experience and provide care in that setting.

